# Designed De Novo α-Sheet Peptides Destabilize Bacterial Biofilms and Increase the Susceptibility of *E. coli* and *S. aureus* to Antibiotics

**DOI:** 10.3390/ijms25137024

**Published:** 2024-06-27

**Authors:** Tatum Prosswimmer, Sarah E. Nick, James D. Bryers, Valerie Daggett

**Affiliations:** 1 Molecular Engineering Program, University of Washington, Seattle, WA 98195, USA; 2 Department of Bioengineering, University of Washington, Seattle, WA 98195, USA; snick36@uw.edu

**Keywords:** alpha-sheet, biofilm, amyloid, antibiotic susceptibility, *Escherichia coli*, *Staphylococcus aureus*

## Abstract

Biofilm-associated microbes are 10–1000 times less susceptible to antibiotics. An emerging treatment strategy is to target the structural components of biofilm to weaken the extracellular matrix without introducing selective pressure. Biofilm-associated bacteria, including *Escherichia coli* and *Staphylococcus aureus*, generate amyloid fibrils to reinforce their extracellular matrix. Previously, de novo synthetic α-sheet peptides designed in silico were shown to inhibit amyloid formation in multiple bacterial species, leading to the destabilization of their biofilms. Here, we investigated the impact of inhibiting amyloid formation on antibiotic susceptibility. We hypothesized that combined administration of antibiotics and α-sheet peptides would destabilize biofilm formation and increase antibiotic susceptibility. Two α-sheet peptides, AP90 and AP401, with the same sequence but inverse chirality at every amino acid were tested: AP90 is L-amino acid dominant while AP401 is D-amino acid dominant. For *E. coli*, both peptides increased antibiotic susceptibility and decreased the biofilm colony forming units when administered with five different antibiotics, and AP401 caused a greater increase in all cases. For *S. aureus*, increased biofilm antibiotic susceptibility was also observed for both peptides, but AP90 outperformed AP401. A comparison of the peptide effects demonstrates how chirality influences biofilm targeting of gram-negative *E. coli* and gram-positive *S. aureus*. The observed increase in antibiotic susceptibility highlights the role amyloid fibrils play in the reduced susceptibility of bacterial biofilms to specific antibiotics. Thus, the co-administration of α-sheet peptides and existing antibiotics represents a promising strategy for the treatment of biofilm infections.

## 1. Introduction

Amyloidogenic proteins are implicated in over 50 mammalian diseases and are considered inherently pathogenic [1,2,3,4,5,6,7,8,9]. Notably, many bacteria utilize mature amyloid fibrils, termed “functional amyloids”, as structural scaffolds in their extracellular biofilm to protect cells from the surrounding environment [10,11,12,13,14,15]. In both cases, the soluble, oligomeric intermediates that form prior to stable fibrils are the primary toxic species, while insoluble fibrils are nontoxic [1,2,3,4,5,6,7,8,9,16,17,18,19]. Toxic oligomers adopt a nonstandard secondary structure, an α-sheet, that has been observed and characterized in several amyloid proteins in both mammals and bacteria, including the amyloid-β peptide (Alzheimer’s disease), islet amylin polypeptide (type 2 diabetes), CsgA (*Escherichia coli*) and PSMα1 (*Staphylococcus aureus*) [3,8,9,10,12,20,21,22,23]. The α-sheet structure is unique in that each residue is locally helical, but alternation between right- and left-handed conformations results in the formation of an elongated strand [3,22,24]. The conserved nonstandard intermediate structure represents a unique target for inhibiting amyloid formation in the context of both mammalian disease and biofilm-associated infection.

Biofilms are microbe-generated, surface-associated extracellular matrices (ECMs) composed of cells and secreted insoluble extracellular molecules (proteins, polysaccharides, and extracellular DNA) that facilitate cell communication and protect cells from the surrounding environment (i.e., host immune response and antibiotics) [15]. Biofilm cells are significantly less susceptible to antibiotics than free-floating (or planktonic) cells and this reduced antibiotic sensitivity is implicated in the rise of multidrug-resistant bacteria and nosocomial infections [25,26].

Nosocomial infections afflict approximately 15% of all hospitalized patients, and these infections often result in prolonged hospital stays, significant financial burden, and disability in both developed and developing countries [27]. Multidrug-resistant bacteria are implicated in at least 14% of all nosocomial infections in the United States, and this number continues to rise [28,29]. Increased resistance is accompanied by a sharp decline in the development of new antibiotics, which has resulted in a global healthcare crisis [30]. Antibiotic resistance is caused in part by the overuse and misuse of antibiotics, as selective pressure can accelerate conferred resistance [31]. Additionally, some bacterial growth conditions, such as biofilm formation, reduce antibiotic susceptibility. Bacteria within a biofilm are 10–1000 less susceptible to antibiotics due to numerous complex mechanisms including the structural barrier provided by the extracellular matrix (ECM), ECM-sequestered antibiotic-degrading enzymes, and phenotypic/metabolic changes in sessile bacteria orchestrated by cell-to-cell communication [26,32,33,34]. Additionally, cells present in the nutrient-lacking biofilm can exhibit lower growth rates and reduced metabolic activity, contributing to reduced antibiotic susceptibility [26]. Biofilms can foster “persister” cells that can often lead to recalcitrance and the re-establishment of infection, and they are thought to have a significant role in acquired resistance [26].

*Escherichia coli* (*E. coli*) and *Staphylococcus aureus* (*S. aureus*) are robust biofilm-forming bacteria that are often implicated in drug-resistant nosocomial infections. Uropathogenic *E. coli* (UPEC) alone account for approximately 50% of all hospital-acquired infections [35]. Although antibiotic resistance varies between isolates, UPEC strains have exhibited resistance to a wide range of antibiotics including fluoroquinolones, amoxicillin, cephalosporin, and ampicillin [35]. Many *S. aureus* strains have also demonstrated clinical resistance to a vast majority of antibiotics including aminoglycosides, penicillin, tetracyclines, all β-lactam antibiotics, and more [25,36].

To date, many anti-biofilm efforts have focused on the destabilization of the structure of the biofilm ECM, prompting bacteria to return to the planktonic state and resulting in increased sensitivity of the treated biofilm to antibiotics. Biofilm dispersal can be achieved using various matrix-degrading enzymes such as proteases, deoxyribonucleases, and glycoside hydrolases [37,38,39]. Other strategies employ anti-biofilm peptides, peptide mimetic graphene quantum dots, or oligosaccharides against specific protein or extracellular DNA targets in the extracellular polymeric substance [40,41,42]. While these techniques can render biofilms more susceptible to antibiotic treatment, some biofilms have shown resistance to various dispersion agents. Further, small molecule agents can be degraded by bacteria or can rapidly diffuse away from biofilms. Thus, the identification of new extracellular targets, such as amyloid proteins, and the development of novel inhibition or dispersion methods is desirable.

The reduced antibiotic susceptibility of many *E. coli* and *S. aureus* species is partially attributed to the use of mature amyloid fibrils as structural biofilm scaffolds and to mediate the dispersion of chemical or mechanical agents [10,11,12,13,14,15]. *E. coli*, a gram-negative bacterium, produces amyloid fibrils known as curli in its biofilms, which are primarily comprised of the protein CsgA [11,43]. In *S. aureus*, a gram-positive bacterium, phenol-soluble modulins (PSMs) aggregate to form amyloid fibrils that fortify the biofilm matrix [12,13,14]. PSMα1 is the dominant component of the mature fibrils [13]. 

As mentioned above, a nonstandard α-sheet structure has been demonstrated in the oligomeric intermediates of both CsgA and PSMα1 [10,12]. To characterize this structure, de novo hairpin peptides composed of alternating L- and D-chirality amino acids that adopt a stable α-sheet structure were produced to specifically target and bind to α-sheet oligomers [3,8,9,10,12,20,21,22,23,24]. These α-sheet peptides are denoted “AP” for “Alternating Peptide”, which indicates alternating L- and D-amino acid templating. Notably, APs inhibit fibrilization and oligomer-associated toxicity of amyloid proteins regardless of their native structure [8,9,10,12,20,21,23]. Further, APs exhibit no specificity toward the monomeric and fibrillar forms of amyloid species, supporting the significance of the α-sheet structure in binding and inhibition [9,44]. 

Previous studies demonstrate that by inhibiting amyloid fibrilization, APs can weaken bacterial biofilms and significantly reduce biofilm cell density in various bacteria, including *E. coli* and *S. aureus* [10,12]. These findings are visually supported by electron microscopy experiments that depict the complete, or near-complete, inhibition of biofilm and amyloid formation by APs in multiple bacteria [10,12,20]. Pertinent to the studies presented here, α-sheet peptides inhibit the aggregation and amyloid formation of both PSMα1 and CsgA in vitro [10,12]. Interestingly, amyloid and biofilm inhibition by APs does not cause cell death, potentially eliminating the concern of selective pressure leading to acquired resistance [10]. Instead, amyloid inhibition facilitates a shift in cells from the biofilm to the planktonic state, rendering them more susceptible to antibiotics [10]. In a previous paper, we showed that AP401 significantly increased the susceptibility of uropathogenic *E. coli* to gentamicin [10]. Here, we expand on our previous work to investigate the ability of AP90 and AP401, two 23-residue α-sheet peptides with identical sequences but opposite chirality at each residue, to increase the susceptibility of *E. coli* and *S. aureus* to a wide range of antibiotics. *E. coli* and *S. aureus* were selected as representative gram-negative and gram-positive species, respectively, and peptide concentrations were chosen based on efficacy in preliminary work. Our goal here is to evaluate the effect of the co-administration of the α-sheet peptides with a range of antibiotics on biofilm susceptibility.

## 2. Results

### 2.1. E. coli UTI89 and S. aureus MN8 Show Low Biofilm Antibiotic Susceptibility

All experiments used either *E. coli* strain UTI89 [45] or *S. aureus* strain MN8 [12], both of which were derived from clinical isolates. UTI89 and MN8 were selected as clinically relevant representative gram-negative and -positive bacteria, respectively. UTI89 is a well-characterized uropathogenic *E. coli* (UPEC) strain from cystitis and is considered a prototype UPEC isolate [46]. MN8 is a toxic shock strain isolated from the urogenital tract.

We first conducted experiments to quantify the susceptibility of mature *E. coli* UTI89 and *S. aureus* MN8 biofilms to five antibiotics: amoxicillin (an amino-penicillin effective against many uropathogenic *E. coli* and *Staphylococcus* species [47]), ciprofloxacin (a broad spectrum fluroquinolone active against both gram-positive and gram-negative bacteria [48]), erythromycin (a macrolide primarily effective against gram-positive bacteria [49]), gentamicin (an aminoglycoside primarily effective against gram-negative bacteria [50]), and vancomycin (a tricyclic glycopeptide used against gram-positive bacteria [51]). These antibiotics were selected to cover a range of antibiotic classes and to target both gram-negative and -positive bacteria. A diverse panel of antibiotics was to chosen to demonstrate that any effects of the peptides on antibiotic susceptibility are not antibiotic-specific. We tested four concentrations per antibiotic (100 µg/mL, 300 µg/mL, 500 µg/mL, and 1 mg/mL) and compared the total colony forming units (CFUs) to the non-antibiotic control condition to measure the decrease in CFUs upon antibiotic addition after 24 (*S. aureus*) or 48 (*E. coli*) total hours. The percentage decrease for each antibiotic condition was calculated from the UTI89 or MN8 CFUs for the non-antibiotic control and the CFUs with antibiotics: percentage decrease = ((CFU_control_ − CFU_abx condition_)/(CFU_control_)) × 100%. Low biofilm antibiotic susceptibility was observed for both bacteria as measured by CFU reductions (Table 1). 

*E. coli* exhibited the highest susceptibility to gentamicin at each of the tested concentrations (4 log or 99.99% CFU reduction at 1000 µg/mL) and showed very little susceptibility to the other antibiotics (Table 1). We predicted that erythromycin and vancomycin would have a minimal effect on *E. coli*, as they are primarily effective against gram-positive bacteria. However, the lack of effect by ciprofloxacin and amoxicillin indicates that UTI89 biofilms exhibit reduced antibiotic susceptibility to multiple drugs.

*S. aureus* exhibited the highest susceptibility to vancomycin at each concentration, although a CFU reduction of 1 log or greater was not observed for any antibiotic combination (Table 1). The highest CFU reduction (88%) was observed at 300 µg/mL, with similar reductions of 86 and 87% observed for 500 or 1000 µg/mL, respectively. Ciprofloxacin was also effective against *S. aureus*, resulting in an 81% reduction at 1000 µg/mL (Table 1). *S. aureus* was minimally susceptible to amoxicillin, erythromycin, and gentamicin with the highest percentage CFU reduction of only 63% for 1000 µg/mL gentamicin (Table 1). As gentamicin is primarily effective against gram-negative bacteria, we expected it to have little effect against *S. aureus*. However, the lack of susceptibility to amoxicillin and erythromycin indicates that the MN8 biofilms are multidrug-resistant. Additionally, the decline in CFU percentage reduction at higher concentrations for some antibiotics (amoxicillin, ciprofloxacin, and erythromycin) is attributed to the fact that they were not fully soluble in the media at concentrations above 500 µg/mL.

Based on these results, an antibiotic concentration of 300 µg/mL was used for the following studies incorporating our α-sheet peptides. This concentration was selected because a reduction in biofilm formation was observed for all antibiotics and both bacterial species at this concentration. Additionally, this antibiotic concentration falls within the range of known minimum biofilm inhibitory concentrations (bMICs) for uropathogenic *E. coli* and methicillin susceptible *S. aureus* (MSSA) for the five antibiotics tested [52,53,54,55,56,57,58,59,60,61]. Rafaque et al. measured the bMICs for 155 uropathogenic *E. coli* strains and reported a range of 128–2048 µg/mL for ciprofloxacin and 64–1024 µg/mL for gentamicin [52]. For MSSA strains, bMICs reported by Pettit et al. were >128 µg/mL for ciprofloxacin and >2048 µg/mL for gentamicin and vancomycin [55,56]. Similarly, Mandell et al. observed bMICs for MSSA isolates ranging from 10 to 1000 µg/mL for gentamicin and 50 to 1000 for vancomycin [55]. The range of bMICs reported in the literature reflects the variation in biofilm antibiotic susceptibility between strains. However, all reports indicate reduced biofilm antibiotic susceptibility as observed with the *E. coli* strain UTI89 and the *S. aureus* strain MN8 in this study.

### 2.2. α-Sheet Peptides Inhibit Amyloid Formation and Reduce Biofilm Density

UTI89 and MN8 biofilms were then grown with α-sheet peptides (no antibiotics) to determine their effect on amyloid formation and biofilm density. The amyloid dye Thioflavin T (ThT) was used as an indicator of amyloid fibril content as it fluoresces upon binding β-sheet fibrils [62]. As ThT also binds nonspecifically to the bacterial cell surface [12], UTI89 experiments included a non-amyloid forming control mutant strain, UTI89 ∆csgA, as an estimate of nonspecific ThT fluorescence.

**Figure 1:**
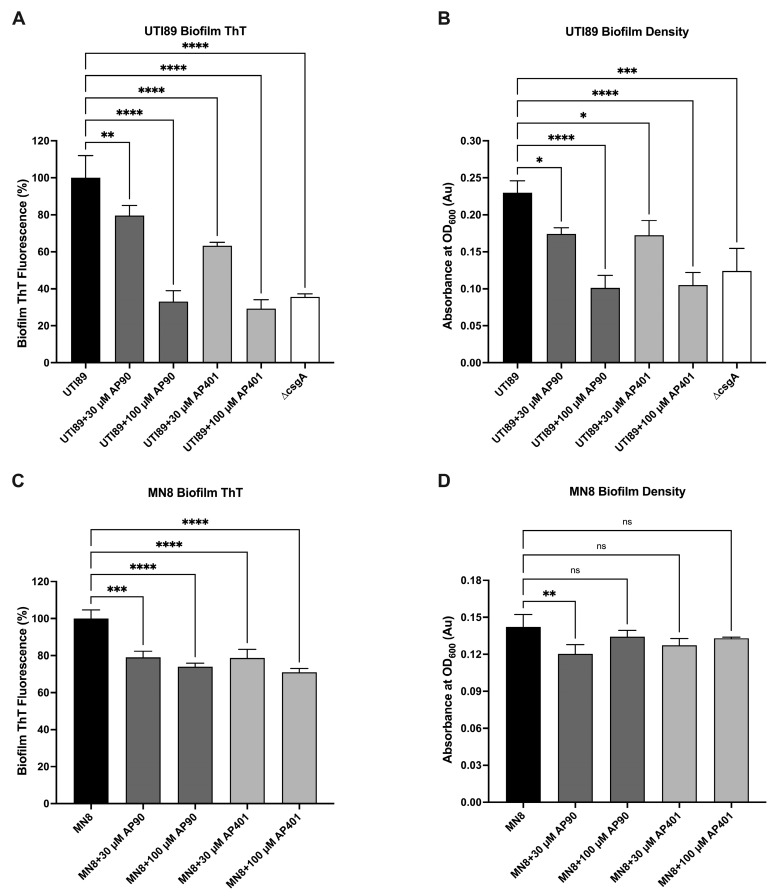
Both α-sheet peptides inhibited amyloid formation as indicated by reduced ThT fluorescence. Biofilm ThT fluorescence values are reported as percent of the average UTI89 or MN8 peptide-free control fluorescence. For UTI89 (**A**), 100 µM AP401 caused the greatest reduction in fluorescence of 70% as compared to the UTI89 control (*p* < 0.0001). Reductions in biofilm formation as quantified by biofilm density measurements (OD_600_) were also observed for both peptides and bacteria. For UTI89 (**B**), 100 µM AP90 and AP401 reduced biofilm formation to the level of the control UTI89 ∆csgA strain. For ThT fluorescence for MN8 (**C**), the 100 µM AP401 condition caused the greatest reduction of 29% as compared to the MN8 control (*p* < 0.0001). For MN8 biofilm density (**D**), the 30 µM AP90 condition significantly reduced biofilm formation. All values are mean and standard deviation for three replicates. Values are mean ± SD or error for three replicates and p-values are indicated as follows: ns = not significant; * *p* < 0.05; ** *p* < 0.01, *** *p* < 0.001, **** *p* < 0.0001.

For UTI89 experiments, a significant reduction in ThT fluorescence was observed for all peptide conditions (Figure 1A). The 100 µM AP90 and AP401 conditions had the largest reductions of 67% and 70%, for AP90 and AP401, respectively, as compared to the UTI89 control (*p* < 0.0001; this p-value indicates that the differences are statistically significant where *p* < 0.05 is the cutoff for determined statistical significance). This reduction was comparable to that of the UTI89 ∆csgA control, indicating complete inhibition of amyloid fibrilization. The difference in ThT reduction between the 100 µM peptide conditions and the UTI89 ∆csgA control was not statistically significant. Critically, the addition of peptides at either concentration did not cause a significant reduction in biofilm ThT fluorescence for the UTI89 ∆csgA control (Supplementary Figure S1A). As the UTI89 ∆csgA strain does not form amyloid, it was expected that the peptides would not affect the baseline ThT fluorescence. For MN8 experiments, a significant reduction in ThT fluorescence was also observed for all peptide conditions (Figure 1C). The 100 µM AP401 condition showed the greatest reduction with a 29% fluorescence reduction as compared to the MN8 control (*p* < 0.0001). 

The optical density (OD_600_) of the biofilms was quantified as a measure of the mass of the biofilms. For UTI89, all peptide conditions caused a significant reduction in biofilm density (Figure 1B). The 100 µM AP90 and AP401 conditions caused a 56% and 54% reduction in biofilm density (*p* < 0.0001), for AP90 and AP401, respectively, as compared to the UTI89 control. This is comparable to the reduction seen for UTI89 ∆csgA. For MN8, a small yet statistically significant reduction in biofilm density was observed only for the 30 µM AP401 condition (*p* = 0.008) (Figure 1D). These data indicate that the α-sheet peptides inhibit amyloid formation as indicated by ThT fluorescence, and that this inhibition correlates to a reduction in biofilm material as measured by optical density. This conclusion is supported by previous studies conducted with PSMα1 and α-sheet peptides in vitro in which the α-sheet peptides inhibited the aggregation and amyloid fibril formation of PSMα1 [12]. Furthermore, the mechanism of action of the α-sheet peptides is the same in both bacterial systems via binding to the α-sheet oligomers of PSMα1 and CsgA to prevent amyloid formation.

**Figure 2:**
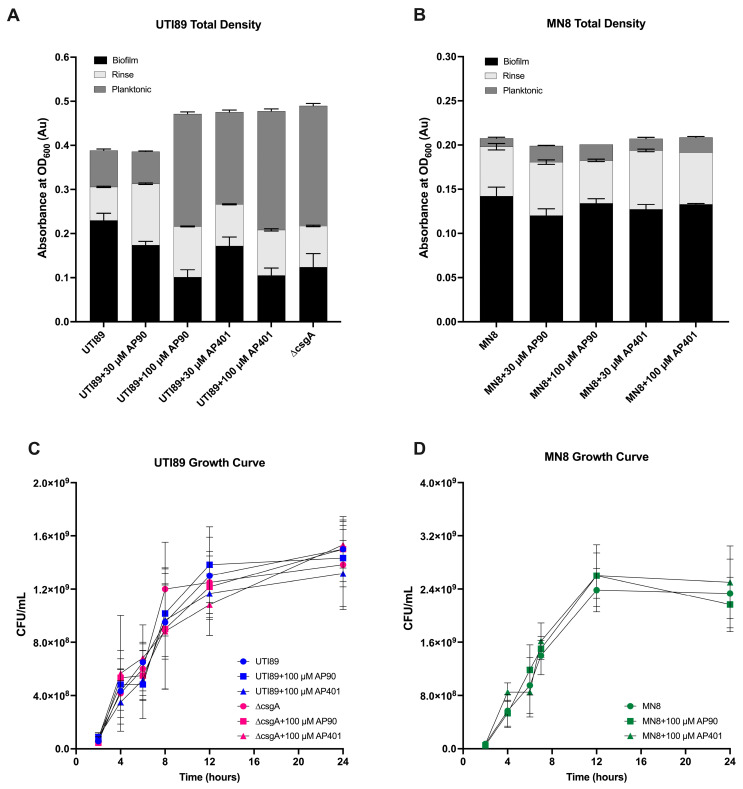
(**A**) For UTI89, the peptides caused a reduction in biofilm density but an increase in planktonic cell density, such that there is not a reduction in overall density. The same effect on overall density is observed for MN8 (**B**) where the total density remains constant with the addition of peptides. All values are mean and standard deviation for three replicates. Growth curves for (**C**) UTI89 and UTI89 ∆csgA strain and (**D**) MN8 in the presence of α-sheet peptides at 100 µM showed that the peptides had no effect on growth rate. All values are mean and standard deviation for three replicates.

Varied efficacy by the two peptides as well as a concentration-dependent response was observed for both bacterial strains. The density of cells in the planktonic state and in the PBS rinse were also quantified with OD measurements (Figure 2A,B). As shown in Figure 1B,D, the addition of the peptides caused a reduction in biofilm mass. Concomitantly, the addition of the peptides caused an increase in the planktonic cell density such that the total density did not decrease, as previously shown for UTI89 [10]. Next, the addition of the peptides did not cause a reduction in the total cell density for the UTI89 ∆csgA control (Supplementary Figure S1B). Finally, UTI89 and MN8 growth curves were performed with growth measured by CFUs in the presence of peptide (Figure 2C,D). The peptides did not affect the growth rates of UTI89, UTI89 ∆csgA, or MN8 in the presence of 100 µM AP90 or AP401 as compared to cells grown in an equal volume of water. This indicates that the peptides did not affect the growth of the cells, only the formation of the mature amyloid.

### 2.3. Curli Fibril Inhibition by AP90 and AP401 Render E. coli More Susceptible to Antibiotics

**Figure 3:**
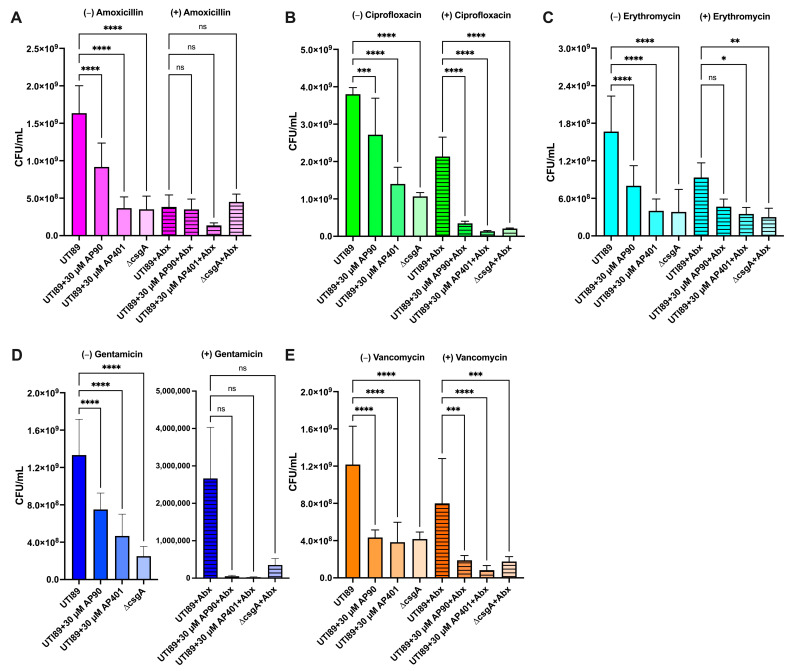
Both synthetic α-sheet peptides inhibited biofilm formation for the non-antibiotic and antibiotic conditions for the *E. coli* strain UTI89. Biofilm formation was quantified as CFU/mL for UTI89 biofilms without (plain bars) and with antibiotics (striped bars) after 48 h. Antibiotics at 300 µg/mL were added after 42 h of growth and biofilm formation was measured for (**A**) gentamicin, (**B**) ciprofloxacin, (**C**) vancomycin, (**D**) amoxicillin, and (**E**) erythromycin. Values are mean ± SD or error for three replicates and p-values are indicated as follows: ns = not significant; * *p* < 0.05; ** *p* < 0.01, *** *p* < 0.001, **** *p* < 0.0001. There is not a statistically significant reduction in CFUs between the peptide conditions and the ∆csgA control for all non-antibiotic and antibiotic conditions.

After establishing the susceptibility of mature biofilms to the antibiotics (Table 1) and determining the effect of the α-sheet peptides on curli fibril and biofilm inhibition (Figure 1), we tested the effect of the co-administration of the α-sheet peptide inhibitors and antibiotics on antibiotic susceptibility. For each *E. coli* antibiotic condition, we compared the susceptibility of the UTI89 strain to the non-amyloid forming control mutant strain, UTI89 ∆csgA. Because our hypothesis is contingent on the idea that curli fortifies the biofilm and reduces antibiotic susceptibility, we predicted that the CsgA knockout strain, UTI89 ∆csgA, would be more susceptible than UTI89 to each antibiotic without α-sheet peptide treatment. This was the case for all antibiotics tested except for amoxicillin where a comparable effect was observed (Figure 3A–E). 

**Figure 4:**
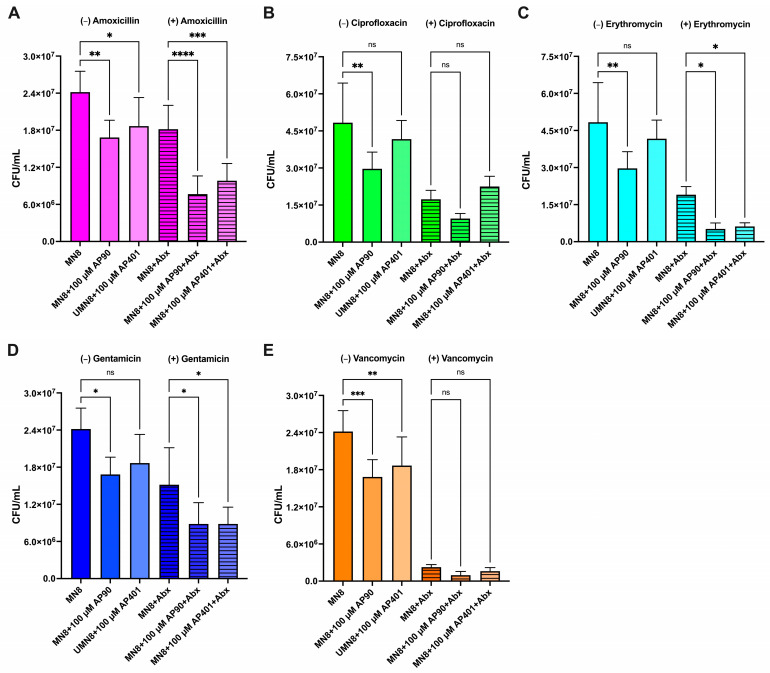
The α-sheet peptides inhibited *S. aureus* biofilm formation for the non-antibiotic and antibiotic conditions to varying extents. Biofilm formation was quantified as CFU/mL for MN8 biofilms without (plain bars) and with antibiotics (striped bars) after 24 h of growth. Antibiotics at 300 µg/mL were added after 18 h of growth and biofilm formation was measured for (**A**) vancomycin, (**B**) erythromycin, (**C**) ciprofloxacin, (**D**) amoxicillin, and (**E**) gentamicin. Values are mean ± SD or error for three replicates and *p*-values are indicated as follows: ns = not significant; * *p* < 0.05; ** *p* < 0.01, *** *p* < 0.001, **** *p* < 0.0001.

Comparing the antibiotic susceptibility of wild-type UT189 treated with α-sheet peptides to the antibiotic susceptibility of UTI89 ∆csgA also confirmed the extent of curli inhibition. Notably, in cases where UTI89 ∆csgA is not more susceptible to an antibiotic than UTI89 treated with α-sheet peptides, we can deduce that biofilm fortification by curli does not play a significant role in *E. coli* antibiotic resistance. In these cases, we would not predict that our peptides would have a large effect on antibiotic susceptibility. For each peptide and antibiotic combination, we presented the CFU counts (Figure 3 and Figure 4) as these values demonstrate the magnitude of the effect of the peptides and the antibiotics. The percentage decrease for each antibiotic condition was calculated from the UTI89 or MN8 CFUs for the non-antibiotic control and the CFUs with antibiotics as previously defined. Our findings are discussed below and summarized in Table 2. 

#### 2.3.1. UTI89 Susceptibility: Gentamicin

The application of gentamicin caused the greatest reduction in UTI89 biofilm CFUs of the five antibiotics tested. As previously demonstrated for the non-antibiotic conditions, the addition of both peptides caused a statistically significant reduction in biofilm CFUs and biofilm OD_600_ (Figure 1B) as compared to UTI89 (*p* < 0.0001) (Figure 3A). Exposure to gentamicin reduced UTI89 biofilm CFUs in the case of both peptides. The observed effect was comparable to that of the UTI89 ∆csgA strain that does not form amyloid fibrils. Of note, there was no statistically significant difference between the peptide conditions and the UTI89 ∆csgA strain, even in cases where the peptide conditions caused a CFU reduction beyond that of the knockout strain. These data indicate that amyloid formation contributes significantly to UTI89 biofilm gentamicin susceptibility (Figure 3A). Both AP90 and AP401 rendered UTI89 more susceptible to gentamicin, resulting in 99.99% CFU reductions as compared to the UTI89 non-antibiotic condition for both peptides (Figure 3A).

#### 2.3.2. UTI89 Susceptibility: Ciprofloxacin

For the non-antibiotic conditions, the addition of each peptide caused a statistically significant reduction in biofilm CFUs, with AP401 causing a greater reduction (*p* = 0.0020 for AP90 and *p* < 0.0001 for AP401) (Figure 3B). A significant CFU reduction was again observed for both α-sheet peptides as compared to the UTI89 strain for ciprofloxacin-treated conditions (*p* < 0.0001) (Figure 3B). Ciprofloxacin applied to UTI89 biofilms resulted in a 44% reduction in CFUs as compared to the non-antibiotic UTI89 condition. (Figure 3B). UTI89 ∆csgA exhibited a 95% CFU reduction upon ciprofloxacin treatment, indicating that amyloid incorporation into UTI89 biofilms has a critical role in establishing susceptibility to the antibiotic (Figure 3B). When grown with AP90 and AP401, the UTI89 CFU reduction from the UTI89 non-antibiotic control increased to 91% and 96%, respectively (Figure 3B). The α-sheet peptide inhibition of curli formation made UTI89 more susceptible to ciprofloxacin, and AP401 increased susceptibility to that of the non-amyloid forming control strain, UTI89 ∆csgA.

#### 2.3.3. UTI89 Susceptibility: Vancomycin

For the vancomycin conditions, both peptides significantly reduced biofilm formation as compared to UTI89 (*p* = 0.0019 for AP90 and *p* = 0.0002 for AP401) and reduced biofilm formation to levels comparable to UTI89 ∆csgA (Figure 3C). Vancomycin exposure led to a 32% reduction in CFUs in UTI89 biofilms and 86% reduction in UTI89 ∆csgA biofilms (Figure 3C). Although vancomycin is primarily used against gram-positive bacteria, the discrepancy in CFU reduction between UTI89 and UTI89 ∆csgA suggests that curli inhibition may result in increased vancomycin susceptibility. Indeed, AP90 and AP401 increased UTI89 CFU reductions to 85% and 93%, respectively (Figure 3E). The incubation of UTI89 with AP401 resulted in a 60% greater biofilm reduction by vancomycin than with no peptide. These data are notable in that they demonstrate that amyloid fibril inhibition can improve susceptibility to a wide range of antibiotics, independent of the antibiotic’s specific mechanism of action. 

#### 2.3.4. UTI89 Susceptibility: Amoxicillin

For the non-antibiotic conditions, the addition of both peptides significantly reduced biofilm CFUs as compared to the UTI89 strain with AP401 showing a reduction equal to that of the UTI89 ∆csgA control strain (*p* < 0.0001 for all) (Figure 3D). For the antibiotic conditions, AP401 caused a non-significant reduction in biofilm formation (Figure 3D). UTI89 and UTI89 ∆csgA exhibited a 77% and 72% reduction in CFUs, respectively, when exposed to 300 µg/mL amoxicillin, suggesting that curli formation does not have a significant effect on biofilm susceptibility to this antibiotic (Figure 3D). We therefore predicted that our designed peptides would not have a large effect on clearance by amoxicillin. Interestingly, the CFU fold reduction of UTI89 increased to 92% when incubated with AP401 (Figure 3D). This indicates that UTI89 is more susceptible to amoxicillin with the co-administration of AP401. In contrast, AP90 had no effect on UTI89 susceptibility to amoxicillin. 

#### 2.3.5. UTI89 Susceptibility: Erythromycin

For the erythromycin conditions, AP401 significantly reduced biofilm CFUs to the level of the UTI89 ∆csgA control strain (*p* = 0.028) (Figure 3E). Erythromycin caused a 44% CFU reduction to UTI89 biofilms and an 82% CFU reduction to UTI89 ∆csgA (Figure 3E). When grown with AP90 or AP401, UTI89 CFU reductions increased to 72% and 79%, respectively (Figure 3E). These CFU reduction values are relatively low even with significant amyloid inhibition by AP90 and AP401 as seen in Figure 1A, but this was expected since erythromycin is not active against gram-negative bacteria. 

### 2.4. AP90 and AP401 Increase S. aureus Biofilm Susceptibility to Antibiotics

#### 2.4.1. MN8 Susceptibility: Vancomycin

For the non-antibiotic conditions, both peptides caused a significant reduction in CFUs (*p* = 0.001 and *p* = 0.0042 for AP90 and AP401, respectively) (Figure 4A). For the vancomycin conditions, incubation with AP90 and AP401 led to a non-significant reduction in biofilm CFUs (Figure 4A). Vancomycin reduced MN8 biofilm CFUs by 91%, and AP90 and AP401 successfully increased susceptibility further, corresponding to CFU reductions of 96% and 93%, respectively (Figure 4A). These data confirm that MN8 amyloid formation reduced biofilm susceptibility to vancomycin and that the amyloid-inhibiting peptides improved antibiotic susceptibility.

#### 2.4.2. MN8 Susceptibility: Erythromycin

The addition of erythromycin to mature MN8 biofilms caused a 61% reduction in biofilm CFUs (Figure 4B). For the non-antibiotic conditions, only AP90 caused a significant reduction in CFUs (*p* = 0.0012) (Figure 4B). For the antibiotic conditions, both peptides caused a significant reduction in CFUs (*p* = 0.02 and *p* = 0.035 for AP90 and AP401, respectively) with reductions of 89% for AP90 and 87% for AP401 (Figure 4B). These increases in CFU reduction suggest that amyloid formation is a factor in the reduced MN8 biofilm susceptibility to erythromycin. 

#### 2.4.3. MN8 Susceptibility: Ciprofloxacin

For the non-antibiotic conditions, both peptides caused a reduction in biofilm formation, with a larger effect observed with AP90 (*p* = 0.0016) (Figure 4C). For the ciprofloxacin conditions, only AP90 caused a reduction in CFU formation (Figure 4C). The addition of ciprofloxacin caused a 64% reduction in CFUs for the MN8 control while the AP90 and AP401 conditions resulted in 80% and 53% reductions, respectively (Figure 4C). The increase in CFU reduction with AP90 indicates that amyloid formation contributed to but was not the primary factor in biofilm susceptibility to ciprofloxacin. Additionally, the difference in CFU change between AP90 and AP401 suggests that peptide chirality played a role in efficacy. 

#### 2.4.4. MN8 Susceptibility: Amoxicillin

For the non-antibiotic conditions, both peptides caused a significant reduction in biofilm formation as compared to the MN8 *S. aureus* strain alone, and AP90 had a larger effect than AP401 (*p* = 0.0038 for AP90 and *p* = 0.04 for AP401) (Figure 4D). A significant reduction was also seen for both peptides for the antibiotic conditions (*p* < 0.0001 for AP90 and *p* = 0.001 for AP401) (Figure 4D). When applied to mature MN8 biofilms, amoxicillin caused a 24% reduction in total CFUs (Figure 4D). Incubation with both AP90 and AP401 increased MN8 susceptibility to the antibiotic, resulting in a 68% and 59% CFU reduction, respectively (Figure 4D). Although the peptides increased antibiotic susceptibility, the magnitude of the change likely indicates that the MN8 biofilm susceptibility to amoxicillin is largely independent of amyloid formation in the biofilm. 

#### 2.4.5. MN8 Susceptibility: Gentamicin

Gentamicin was predicted to have a minimal effect on *S. aureus* as it targets aerobic gram-negative bacteria by passing through the gram-negative membrane [50]. Both peptides caused a moderate reduction in biofilm formation as compared to the MN8 strain with gentamicin only (*p* = 0.04 for both peptides) (Figure 4E). When applied to mature MN8 biofilms, gentamicin reduced CFUs by 37% (Figure 4E). Incubation with AP90 and AP401 increased MN8 susceptibility, corresponding to a 63% reduction in biofilm CFUs for both peptides (Figure 4E). 

## 3. Discussion

Increased rates of antibiotic resistance pose a global threat, thus there exists a significant need to develop novel methodologies to target microbial infections, particularly those that are biofilm-associated. The ongoing emergence of multidrug-resistant strains and the use of some drugs only as antibiotics as a last resort disincentivizes the development of novel antibiotics. Approaches like the strategy presented here are advantageous as they may facilitate the continued use of existing antibiotics for treatment. Here, we present a strategy to increase the susceptibility of amyloid-forming bacteria to antibiotics without introducing selective pressure as caused by bactericidal compounds and reducing the risk of acquired resistance. We have previously shown that α-sheet peptides inhibit curli fibril formation, increase *E. coli* susceptibility to gentamicin, and improve macrophage clearance [10]. The findings reported here expand on our previous research by demonstrating that α-sheet peptides reduce biofilm cell density as reported by CFU quantification (Figure 3 and Figure 4) and OD_600_ (Figure 1B,D), and are effective at increasing antibiotic susceptibility of both gram-negative (*E. coli*) and gram-positive (*S. aureus*) bacteria to multiple classes of antibiotics (Table 2) with varying mechanisms of action. The results reported here suggest that our peptides may be effective against additional amyloid-producing bacteria. 

Although our α-sheet peptides proved effective at increasing antibiotic susceptibility of clinically derived strains of both *E. coli* and *S. aureus*, there were key differences in the response of the bacteria to amyloid inhibition by APs. A higher peptide concentration was required to achieve a significant increase in *S. aureus* antibiotic susceptibility (100 µM vs. 30 µM in *E. coli*), and the observed differences likely arise because *E. coli* is a gram-negative bacterium while *S. aureus* is gram-positive. Gram-positive bacteria are encompassed by thick layers of peptidoglycan, while gram-negative bacteria have much thinner peptidoglycan cell walls surrounded by an outer membrane composed of lipopolysaccharide [63]. Variations in the cell surface may differentially affect the ability for the de novo α-sheet peptides to access the growing amyloid located on the cell surface. The observed differences may also be due to variations in the amount of amyloid precursor secreted by and incorporated into the biofilm of *E. coli* and *S. aureus*. 

We also observed discrepancies in the relative potency of AP90 and AP401 in the two bacteria. Previously, we found that AP401 is a more potent inhibitor of curli formation than its structural isomer, AP90. AP90 and AP401 have the same amino acid sequence, but every amino acid has the opposite chirality, and we hypothesized that the potency of AP401 in *E. coli* was due to the presence of D-amino acids in the hairpin turn resulting in increased stability to proteases. The results presented here suggest that AP401 is not only a more potent inhibitor of curli formation, but that this elevated inhibition also translates to a larger effect on antibiotic susceptibility. Interestingly, there was a smaller potency difference between AP90 and AP401 in *S. aureus* with AP90 causing a greater effect for four antibiotics. D-amino acids are frequently incorporated into gram-positive cell walls which are composed of thick peptidoglycan layers [64]. Because *S. aureus* utilizes D-amino acids in its cell wall, the bacteria may also produce proteases that are designed to cleave the peptide bond between D-amino acids. 

Our designed peptides improved bacterial susceptibility to varying extents for each antibiotic. These observed variations can be attributed both to the differences between the mechanism of action of each antibiotic, as well as to the mechanism that governs the bacterial susceptibility to the antibiotic. AP90 and AP401 are amyloid inhibitors, and our hypothesis is contingent on biofilm amyloid formation having a significant effect on antibiotic susceptibility. However, AP90 and AP401 may not have any effect on a bacterium’s susceptibility if the mechanism of reduced susceptibility is unrelated to biofilm fortification by amyloid fibrils. The largest increase in antibiotic susceptibility upon peptide administration was seen with gentamicin for *E. coli* and vancomycin for *S. aureus*, indicating that amyloid formation was contributing significantly to antibiotic susceptibility in these cases. 

Previous studies have identified several other compounds with anti-amyloid activity against bacterial biofilms, including peptides, proteins, curlicides, graphene quantum dots, and polyphenols [43,65,66,67]. However, in some cases, such as for the plant flavonoids luteolin, myricetin and quercetin, biofilm inhibition was seen in some bacteria (*E. coli* and *S. aureus*) while an increase or no change in biofilm formation was observed in other bacteria (*Pseudomonas aeruginosa*) [68]. Additionally, while some compounds have successfully inhibited biofilm formation, most compounds have not been tested with antibiotics, or have been shown to increase antibiotic resistance. In one study, the anti-amyloidogenic polyphenol epigallocatechin gallate (EGCG) caused reduced susceptibility of *S. aureus* to vancomycin, oxacillin, and ampicillin [69]. Another study found that in some conditions, EGCG administration promoted biofilm formation in *P. aeruginosa* and increased antibiotic resistance to tobramycin [70]. A third study reported that the co-administration of EGCG and tobramycin had a moderate effect on wildtype *P. aeruginosa* biofilm minimum bactericidal eradication concentrations but a larger effect when the functional amyloid fibril Fap was overexpressed [71]. Thus, it is critical to test the efficacy of potential bacterial amyloid inhibitors in combination with antibiotics, as performed in this study. 

## 4. Materials and Methods

### 4.1. Peptide Synthesis

Synthetic α-sheet peptide inhibitors were designed in silico as previously described [8,10,12,23], using backbone dihedral angle constraints derived from MD simulations [72,73] and synthesized as described by Bleem et al. [10]. Briefly, peptides contain two α-strands of seven residues each, with amino acids alternating sequentially between L-conformation and D-conformation in each of the strands. The α-strands are connected by a five-residue turn comprised of all L-amino acids (AP90) or all D-amino acids (AP401), which gives the peptide a hairpin shape. Finally, the tail of each strand consists of a Gly and an Arg residue, followed by acetyl and amide caps at the N- and C-terminus, respectively. Peptides were assembled by solid phase peptide synthesis on Rink amide resin with Fmoc chemistry and HBTU activation. Peptides were cleaved from the resin and side chain deprotected by TFA/TIPS/H_2_O (95:2.5:2.5) and precipitated by cold ether. Crude peptides were purified to >95% by RP-HPLC using 5 μM C12 or C18 100 Å columns (Phenomenex; Torrance, CA, USA) and atomic masses were confirmed by electrospray mass spectrometry on a Bruker Esquire Ion Trap (Bruker; Billerica, MA, USA). Sequences for the two α-sheet designs described in this study (AP90 and AP401) are listed in Table 3. All peptides were lyophilized after purification and stored at −20 or −80 °C until use. 

### 4.2. E. coli Biofilm Growth

A uropathogenic clinical isolate strain, UTI89 [45], and a control strain with a chromosomal deletion of the *csgA* gene, UTI89 ∆csgA [43], were used for all *E. coli* experiments. Overnight cultures were grown in 25 g/L Luria Broth (LB; Miller, Thermo Fisher Scientific; Waltham, MA, USA) for 16–18 h at 37 °C with shaking (180 rpm). Cultures were then “refreshed” by replacing 5 mL of culture with 5 mL fresh LB medium and grown for an additional three hours to ensure bacteria were in the exponential phase. Overnight cultures were then diluted to an optical density (OD_600_) of 0.1 (~8 × 10^7^ cells/mL) in YESCA broth supplemented with 4% DMSO (Corning; Glendale, AZ, USA), medium known to promote increased curli formation [74]. Lyophilized peptide stock was dissolved in water and concentrations were determined by Nanodrop^TM^ (Thermo Fisher Scientific; Waltham, MA, USA). Diluted bacteria culture (180 µL) was plated with 20 µL peptide (or water, in the case of controls) and aliquoted in triplicate into wells of a sterile, clear 48-well polystyrene plate (Corning; Glendale, AZ, USA). The final peptide concentration was 0, 30 µM (1.2 pg/CFU), or 100 µM (4 pg/CFU). Plates were covered, sealed in a plastic bag, and statically grown at 26 °C for 48 h. 

### 4.3. S. aureus Biofilm Growth

*S. aureus* MN8 (clinical isolate; urogenital tract; [75]) was grown for 16–18 h in 10 g/L trypticase soy broth (TSB; Becton, Dickinson and Company; Sparks, MD) at 37 °C with shaking (180 rpm). Overnight cultures were “refreshed” by replacing 5 mL of culture with 5 mL of fresh TSB medium and grown for an additional six hours. Cells were then spun down and re-suspended in peptone-NaCl-glucose (PNG) media (Thermo Fisher Scientific; Waltham, MA, USA) [14]. Resuspended cells were diluted to an optical density of 0.1 (OD_600_). Peptides were prepared as described above. Diluted bacteria culture (180 µL) was plated with 20 µL peptide (or water, in the case of controls) and aliquoted in triplicate into wells of a sterile, clear 48-well polystyrene plate (Corning; Glendale, AZ, USA). The final peptide concentration was 0, 30 µM (1.2 pg/CFU), or 100 µM (4 pg/CFU). Plates were covered, sealed in a plastic bag, and grown at 37 °C for 24 h with shaking (250 rpm). 

### 4.4. Thioflavin T (ThT) Assay, Biofilm and Total Density Measurements

After 24 or 48 h of growth for *S. aureus* or *E. coli*, respectively, planktonic cells and medium were removed and biofilms were rinsed once with 250 μL PBS. Planktonic cells were spun down and resuspended in PBS, and the optical density of both planktonic and rinse samples was determined at 600 nm to estimate “planktonic” and “rinse” cell densities. The PBS rinse solution was removed from the wells and biofilms were resuspended in 200 μL of 20 μM ThT in PBS (Sigma- Aldrich; St. Louis, MO, USA). Biofilms were homogenized by vigorous pipetting (30× per well), 3 min of sonication, and 1 min on a plate shaker. Next, 100 μL of each biofilm suspension was transferred to a black-walled, clear-bottom 96-well plate for measurements in a plate reader (PerkinElmer; Waltham, MA, USA). ThT fluorescence was measured at 438/495 nm as a proxy for amyloid formation, and biofilm absorbance was measured at 600 nm to estimate bacterial cell density. Fluorescence measurements were corrected for nonspecific fluorescence by subtracting the background intensity of identical samples without bacteria. Biofilm ThT fluorescence values are reported as percent of the average MN8 or UTI89 peptide-free control fluorescence. All values are mean and standard deviation for three replicates. 

### 4.5. Growth Curves

Overnight cultures of UTI89 and UTI89 ∆csgA were grown in 25 g/L Luria Broth (LB; Miller, Thermo Fisher Scientific; Waltham, MA) for 16–18 h at 37 °C with shaking (180 rpm). Overnight cultures of MN8 were grown in 10 g/L TSB. For each condition, 30 µL of the overnight was added to 3 mL of fresh LB or TSB medium supplemented with water or 100 µM AP90 or AP401 (final concentration) and grown at 37 °C with shaking (180 rpm). At each time point (2, 4, 6, 7 or 8, 12, and 24 h), 200 µL of media was removed, spun down, and resuspended in an equal volume of PBS (Sigma- Aldrich; St. Louis, MO, USA). The samples were then ultra-sonicated for 5 s on ice and diluted in tenfold increments. The serial dilutions were then plated on agar plates (LB agar or TSB agar) using the drop plate method [76]. Six replicates were plated per condition. Colonies were grown for 16 h at 37 °C and CFUs were counted. Total CFUs of the suspensions were calculated using the dilution number and the number of CFUs counted in that dilution.

### 4.6. Antibiotic Susceptibility

Biofilms were grown according to the methods described above. Five antibiotics were tested: amoxicillin (MP Biomedicals: Solon, OH, USA), ciprofloxacin hydrochloride (MP Biomedicals: Solon, OH, USA), erythromycin (Thermo Fisher Scientific; Waltham, MA, USA), gentamicin sulfate (Thermo Fisher Scientific; Waltham, MA, USA), and vancomycin hydrochloride (Thermo Fisher Scientific; Waltham, MA, USA). Antibiotics were dissolved in YESCA (*E. coli*) or PNG medium (*S. aureus*) at a concentration of 900 µg/mL. Next, 100 µL of antibiotic or control (YESCA/PNG media) was added to each well after 42 (*E. coli*) or 18 (*S. aureus*) hours of incubation without disturbing the biofilm for a final well concentration of 300 µg/mL. Following 6 additional hours of biofilm growth (48 or 24 h total), planktonic cells were removed and discarded. The biofilms were rinsed with 250 µL PBS (Sigma Aldrich; St. Louis, MO, USA), and the rinse was discarded. Biofilms were homogenized in 200 µL PBS by vigorous pipetting (30× per well), and the biofilm suspensions were transferred to an Eppendorf tube. The biofilm suspensions were then ultra-sonicated for 5 s on ice and diluted in tenfold increments. The serial dilutions were then plated on agar plates (LB agar or TSB agar) using the drop plate method [76]. Six replicates were plated per condition. Colonies were grown for 16 h at 37 °C and CFUs were counted. Total CFUs of the biofilm suspensions were calculated using the dilution number and the number of CFUs counted in that dilution. The percentage decrease for each antibiotic condition was calculated from the UTI89 or MN8 CFUs for the non-antibiotic control and the CFUs with antibiotics: percentage decrease = ((CFU_control_ − CFU_abx condition_)/(CFU_control_)) × 100%.

### 4.7. Statistics

All statistical significance values reported are calculated with one-way ANOVA with post hoc comparisons as shown and a Bonferroni multiple comparison correction performed in GraphPad Prism Version 10.1.0. A single asterisk indicates a *p*-value less than 0.05, which is the cutoff to be considered statistically significant. Two asterisks indicate greater significance, with a *p*-value less than 0.01. Three asterisks indicate a *p*-value less than 0.001. Four asterisks indicate a *p*-value less than 0.0001. 

## Figures and Tables

**Table 1:** CFU percentage reductions of UTI89 and MN8 biofilms to five antibiotics indicate multidrug resistance. CFU percent reductions for the addition of antibiotics to UTI89 and MN8 biofilms were determined for CFUs measured after 48 (UTI89) or 24 (MN8) hours. The percentage decrease for each antibiotic condition was calculated from the UTI89 or MN8 CFUs for the non-antibiotic control and the CFUs with antibiotics: percentage decrease = ((CFU_control_ − CFU_abx condition_)/(CFU_control_)) × 100%. * Some antibiotics (amoxicillin, ciprofloxacin and erythromycin) are not fully soluble in media at concentrations of 500 µg/mL and 1000 µg/mL.

		Antibiotic Concentration (µg/mL)			
		100	300	500 *	1000 *
*E. coli* UTI89	Amoxicillin	39	78	88	56
	Ciprofloxacin	44	70	79	60
	Erythromycin	47	40	42	48
	Gentamicin	99	99.9	99.99	99.99
	Vancomycin	38	43	47	53
*S. aureus* MN8	Amoxicillin	48	31	26	18
	Ciprofloxacin	40	54	72	81
	Erythromycin	11	45	37	51
	Gentamicin	45	53	59	63
	Vancomycin	59	88	86	87

**Table 2:** Summary of susceptibility results for UTI89 and MN8 co-administration of α-sheet peptides AP90 or AP401 with five antibiotics. All peptide and antibiotic combinations increased biofilm antibiotic susceptibility, although to varying extents. Check marks indicate an increase in antibiotic susceptibility as determined by CFU percentage decrease from the UTI89 or MN8 non-antibiotic control where ✓ = 50–80% reduction, ✓✓ = 81–90% reduction, ✓✓✓ = 91–95% reduction, ✓✓✓✓ = 96–99% reduction, and ✓✓✓✓✓ = >99% reduction.

		Increased Susceptibility?	
		AP90 + abx	AP401 + abx
*E. coli* UTI89	Amoxicillin	✓	✓✓✓
	Ciprofloxacin	✓✓✓	✓✓✓
	Erythromycin	✓	✓
	Gentamicin	✓✓✓✓✓	✓✓✓✓✓
	Vancomycin	✓✓	✓✓✓
*S. aureus* MN8	Amoxicillin	✓	✓
	Ciprofloxacin	✓	✓
	Erythromycin	✓✓	✓✓
	Gentamicin	✓	✓
	Vancomycin	✓✓✓✓	✓✓✓

**Table 3:** Peptide sequences and bacterial strain descriptions. ^a^ “AP” refers to “Alternating Peptide”, which indicates alternating l- and d-amino acid templating. ^b^ l-amino acids are displayed in all upper case; d-amino acids are displayed in lower case and underlined.

Peptide Sequences			
Name ^a^	Sequence ^b^	Description	Source
AP90	Ac-RGEmNlSwMNEYSGWtMnLkMGR-NH2	α-sheet monomer	Hopping et al., 2014 [8]
AP401	Ac-rGeMnLsWmneysGwTmNlKmGr-NH2	α-sheet monomer	Bleem et al., 2017 [12]
*E. coli* Strains			
UTI89		UPEC strain; cystitis isolate	Mulvey et al., 2001 [45]
UTI89 ΔcsgA		UPEC strain; cystitis isolate with chromosomal deletion of *csgA* gene	Cegelski et al., 2009 [43]
*S. aureus* Strain			
MN8		Clinically relevant strain; toxic shock isolate, urogenital tract	Schwartz et al., 2012 [14]

## Data Availability

The data generated and analyzed during this study are included in the body of the paper. Datasets are available from the corresponding author on reasonable request.

## Supplementary Materials

The following supporting information can be downloaded at: https://www.mdpi.com/article/10.3390/ijms25137024/s1.
