# Supplementary material for: Designed De Novo α-Sheet Peptides Destabilize Bacterial Biofilms and Increase the Susceptibility of E. coli and S. aureus to Antibiotics
Source: Int J Mol Sci. 2024 Jun 27;25(13):7024. doi: 10.3390/ijms25137024 (PMC11241457; doi:10.3390/ijms25137024)
Supplement: Supplementary file 1 [file ijms-3029290-supplementary.pdf › ijms-3029290-supplementary.pdf]

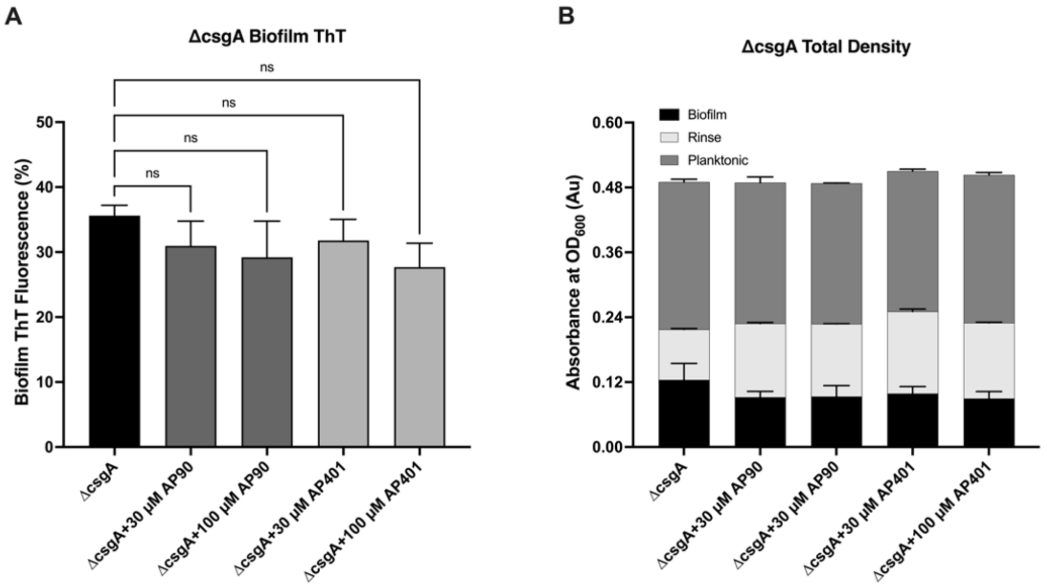

Figure S1. (A) Addition of the peptides to the UTI89 ΔcsgA control did not cause a reduction in ThT fluorescence as the strain does not produce amyloid. Addition of the peptides also did not cause a decrease in the total cell density of the UTI89 and MN8 strains. (**B)** For the UTI89 ΔcsgA control, addition of peptides did not reduce biofilm or planktonic cell density. All values are mean and standard deviation for three replicates.
